# Blood Leukocyte ROS Production Reflects Seminal Fluid Oxidative Stress and Spermatozoa Dysfunction in Idiopathic Infertile Men

**DOI:** 10.3390/antiox12020479

**Published:** 2023-02-14

**Authors:** Matteo Becatti, Gianmartin Cito, Flavia Rita Argento, Eleonora Fini, Alessandra Bettiol, Serena Borghi, Amanda Mannucci, Rossella Fucci, Claudia Giachini, Rita Picone, Giacomo Emmi, Niccolò Taddei, Maria Elisabetta Coccia, Claudia Fiorillo

**Affiliations:** 1 Department of Experimental and Clinical Biomedical Sciences “Mario Serio”, University of Firenze, 50134 Firenze, Italy; 2 Department of Experimental and Clinical Medicine, University of Firenze, 50134 Firenze, Italy; 3 Assisted Reproductive Technology Centre, Careggi University Hospital, 50134 Firenze, Italy

**Keywords:** men infertility, leukocyte ROS production, oxidative stress, sperm dysfunction

## Abstract

A large proportion of infertile men do not receive a clear diagnosis, being considered as idiopathic or unexplained cases due to infertility diagnosis based on standard semen parameters. Particularly in unexplained cases, the search for new indicators seems mandatory to provide specific information. In the etiopathogenesis of male infertility oxidative stress displays important roles by negatively affecting sperm quality and function. In this study, performed in a population of 34 idiopathic infertile men and in 52 age-matched controls, redox parameters were assessed in blood, leukocytes, spermatozoa, and seminal fluid and related to semen parameters. The main findings indicate that blood oxidative stress markers reflect seminal oxidative stress. Interestingly, blood leukocyte ROS production was significantly correlated to sperm ROS production and to semen parameters. Overall, these results suggest the potential employ of blood redox markers as a relevant and adjunctive tool for sperm quality evaluation aimed to preconception care.

## 1. Introduction

It has been recently estimated that, globally, 8–12% of couples suffer from infertility, with the male factor being a primary or contributing cause in approximately 50% of couples. Male infertility is a complex multifactorial pathological condition comprising congenital, acquired, or idiopathic factors [1]. Current standard clinical diagnostics for male factor infertility (sperm count, motility, and morphology) reveal useful information for the initial evaluation of male infertility, but it is not a direct test of fertility [2]. Idiopathic male infertility is assumed to be caused by several factors, including endocrine disruption as a result of environmental pollution, genetic and epigenetic abnormalities, and oxidative stress [1,3].

In particular, the involvement of oxidative stress, a condition characterized by an imbalance between reactive oxygen species (ROS) production and antioxidant defense systems, has been repeatedly invoked in the etiopathogenesis of male infertility [4,5,6,7,8], but contradictory findings have been reported [8,9,10,11,12,13]. ROS display several physiological functions such as the development of sperm fertilization properties, promotion of chromatin compaction in maturing spermatozoa, motility, chemotaxis, sperm capacitation, hyperactivation, acrosome reaction, and oocyte interaction are included [14,15]. Sperm mitochondria, morphologically abnormal spermatozoa, and activated leukocytes in seminal fluid represent the main ROS sources in the male reproductive system [16]. In particular, leukocytes are considered the major contributors to ROS production, being able to generate 1000 times more ROS than spermatozoa [17]. In line with these observations, in the seminal fluid of infertile men signs of oxidative stress (as indicated by higher ROS and lower antioxidant levels) compared to healthy subjects have been repeatedly reported [18,19]. Indeed, it has been estimated that oxidative stress at the seminal level contributes to up to 80% of all infertility diagnoses [8].

Excessive ROS production has been also suggested to affect essential metabolic/functional sperm cell processes [20] and to damage the spermatozoa plasma membrane, which is characterized by a high content of polyunsaturated fatty acids (specific ROS targets) [21,22]. Upon ROS attack, disruption of membrane permeability, and ATP efflux, flagellar movement is impaired. Moreover, due to the loss of most cell organelles and DNA transcription, spermatozoa lack protein expression and vesicular transport [23]: this implies that the depleted intracellular antioxidant enzymes and oxidized plasma membrane proteins/lipids cannot be newly synthesized, hence promoting oxidative stress-induced cellular damage. To this end, seminal fluid is physiologically equipped with enzymatic and non-enzymatic antioxidants [24,25] whose deficiencies lead to detrimental effects on sperm quality and function [26,27]. At the DNA level, ROS-induced DNA damage in human spermatozoa has been correlated with adverse events including reduced fertilization, dysregulated pre-implantation embryo development, recurrent pregnancy loss (RPL), childhood mortality, and high rates of offspring morbidity [27,28,29,30,31].

Currently, oxidative stress evaluation is being increasingly practiced due to the huge body of evidence suggesting its clinical utility [32] particularly in ART [28].

Unfortunately, guidelines on standardized reference values for both pathological ROS levels and total antioxidant capacity (TAC) in seminal fluid are still lacking [19,33]. In our previous studies, we observed that ROS production by peripheral blood leukocytes represents an excellent model to study systemic oxidative stress-related disorders [34,35,36,37]. In the present study, leukocyte ROS production and redox status in the blood and semen of idiopathic infertile men and controls have been assessed. Correlation analyses between redox parameters in blood and semen were assessed. Furthermore, the correlation of semen oxidative status versus semen parameters was analyzed.

## 2. Materials and Methods

### 2.1. Patients

The study was performed in accordance with the Declaration of Helsinki and approved by the Ethical Review Board of the Careggi University Hospital (reference n. 10709 approved on 27 April 2017). The study sample included a total of 34 idiopathic infertile men (aged 34–56 years) without abnormal andrological findings and with abnormal semen parameters belonging to couples with fertile women who attended the IVF Center of the Careggi University Hospital (Florence, Italy) for IVF procedures (IVF or ICSI) and 52 men matched by age, recruited as control group belonging to couples with tubal factor female infertility, defined as normozoospermic according to the World Health Organization 2021 criteria (WHO laboratory manual for the examination and processing of human semen, 2021). All participants gave written informed consent to use the remainder of their semen sample prior to inclusion in the study.

At the baseline visit, men were routinely screened for serology, according to WHO 2021 guidelines including HIV 1/2, hepatitis C virus antibody (HCVab), hepatitis B surface antigen (HbsAg), hepatitis B surface antibody (HbsAb), hepatitis B c antibody (HBcAb), Treponema Pallidum Hemagglutination and Venereal Disease Research Laboratories (TPHA-VDRL), Ab anti-Clamidya Trachomatis, CMV IgM and IgG. The hormonal panel, including follicle-stimulating hormone (FSH), luteinizing hormone (LH), total testosterone (TT), thyroid-stimulating hormone (TSH), and prolactin (PRL), was required. All patients and controls included in the study displayed normal levels of FSH, LH, TT, PRL, and TSH (normal levels of FSH, LH, and TT were considered, respectively, 1.5–8.0 IU/L, 1.8–12 IU/L, and 2.7–18 ng/mL. PRL levels were considered normal between 3.0 and 18 ng/mL and TSH between 0.3 and 5.5 mIU/L) The genetic evaluation was performed, including the karyotype with the examination of microdeletions for chromosome Y and mutation of cystic fibrosis transmembrane conductance regulator (CFTR) genes. All men performed a urine analysis and urethral swab to detect urinary tract infections. The exclusion criteria were leukocytospermia (defined as leukocyte concentration greater than 1 × 10^6^/mL), azoospermia or severe criptozoospermia, history of smoking (>5 years), excessive alcohol consumption (≥15 drinks per week), endocrine disorders, drug intake, and those with varicocele. Additionally, men with significant comorbidities, including cancer, diabetes, obesity, autoimmune disease, gastrointestinal disease, kidney disease, and lung disease, and men seropositive for HIV, HCV, HBsAg, CMV IgM were excluded from the study. Medical anamnesis and physical examination were performed on all patients.

At baseline, semen analysis was completed by following the World Health Organization (WHO) guidelines for semen examination 2021 (WHO laboratory manual for the examination and processing of human semen) [38]. Semen parameters were recorded: volume (ml), pH, sperm concentration (10^6^ per ml), total sperm number (10^6^ per ejaculate), vitality (live spermatozoa, %), progressive motility (PR, %), non-progressive motility (NP, %), immotile spermatozoa (IM, %), total motility (PR + NP, %) and morphology (normal forms, %). For the determination of anti-sperm antibodies in semen, the mixed antiglobulin reaction (MAR) test was used as described elsewhere [39]. A MAR test result (motile spermatozoa with bound particles, %) >50% was considered positive.

### 2.2. Seminal Fluid and Blood Collection

For each patient, semen and peripheral blood samples were obtained. Semen samples were collected on site by masturbation after 2–5 days of sexual abstinence. The ejaculate was allowed to liquefy before measurement (less than 30 min but, in accordance with 2021 WHO core ejaculate examination methods, no longer than 1 h). Ejaculates were then examined with standard procedures according to 2021 WHO guidelines [38]. Seminal plasma was centrifuged at 16,100× *g* for 1 h at 4 °C to remove cellular debris and then assayed for oxidative stress parameters.

Blood samples were collected in Vacutainer tubes containing 0.109 mol/L buffered trisodium citrate (1:10) or EDTA (0.17 mol/L). After centrifugation (1500× *g* for 15 min at 4 °C), aliquots of sodium citrate plasma were used for experiments or stored at −80 °C for further analyses [40].

### 2.3. Intracellular ROS Levels Assessment in Blood Leukocytes and Spermatozoa by Flow Cytometry Analysis

An amount of 100 µL of EDTA-anticoagulated blood samples was suspended in 2 mL of BD FACS Lysing Solution (Becton Dickinson Biosciences, San Jose, CA, USA), gently mixed, and incubated at room temperature in the dark for 15 min. Next, cells were centrifuged (700× *g* for 7 min at 20 °C), the supernatant was discarded, and cells were washed twice in PBS. The evaluation of leukocyte intracellular ROS levels was performed by incubating cells with H2DCF-DA (2.5 µM) (Invitrogen, Carlsbad, CA, USA) in RPMI medium without serum and phenol red for 30 min at 37 °C [41]. After labeling, cells were washed and suspended in PBS and then immediately analyzed using FACSCanto flow cytometer (Becton-Dickinson, San Jose, CA, USA). The sample flow rate was adjusted to about 1000 cells/s. For a single analysis, the fluorescence properties of at least 20,000 events were collected per sample. The individual cell subpopulations were gated using their distinctive forward-scatter and side-scatter properties. Moreover, cell viability was evaluated by flow cytometry with propidium iodide staining and it was found to exceed 95%. Data were analyzed using BD FACSDiva software (Becton-Dickinson, San Jose, CA, USA).

Spermatozoa intracellular ROS production was assessed using the same discussed method. Samples were centrifuged (700× *g* for 8 min at 20 °C), the supernatant was stocked at −80 °C for further redox analyses, while sperm cells were incubated with H2DCF-DA (2.5 µM) (Invitrogen, Carlsbad, CA, USA) for 30 min at 37 °C as previously described and then analyzed by FACSCanto flow cytometer (Becton-Dickinson, San Jose, CA, USA).

### 2.4. Lipid Peroxidation Estimation in Blood and Seminal Plasma

Lipid peroxidation was assessed in both blood and seminal plasma using ALDetect Lipid Peroxidation Assay Kit (BML-AK170 ASSAY, ENZO Life Sciences), designed to measure malondialdehyde (MDA) in combination with 4-hydroxyalkenals in methanesulfonic acid. Briefly, 200 µL of undiluted plasma sample or standard curve point were added to 650 µL of N-methyl-2-phenylindole prepared in a solution of acetonitrile/methanol (3:1). Immediately, 150 µL of methanesulfonic acid containing 34 uM Fe (III) were also added to each sample starting the reaction. Samples were incubated at 45 °C for 4 h, then centrifuged twice at 15,000× *g* at 4 °C and transferred in a 96 multiwell. MDA (final concentration 75 uM) was used as standard. Absorbance was measured at 586 nm in a Microplate Fluorometer (Biotek Synergy H1). Results were expressed in terms of MDA equivalent (nmol/mL).

### 2.5. Total Antioxidant Capacity (TAC) Estimation in Blood and Seminal Plasma

TAC value in blood and seminal plasma was estimated using ORAC (oxygen radical absorbance capacity) method. This assay is based on the intensity fluorescence decay of a fluorescent probe, fluorescein, consequent to its oxidation by free radical species (particularly peroxyl radical), generated after the thermal decomposition of 2,2′-azobis(2-amidinopropane) dihydrochloride (AAPH) azo-compound. A fluorescein solution (6 nM) prepared daily from a 4 µM stock in 75 mM sodium phosphate buffer (pH 7.4), was used. Trolox (250 µM final concentration), a water-soluble analog of E vitamin, was used as standard. An amount of 70 µL of each plasma sample (diluted 1:200 in sodium phosphate buffer) or standard curve point was pre-incubated in each well with 100 µL of fluorescein for 30 min at 37 °C and then 50 µL of AAPH solution (19 mM final concentration) was added starting the reaction [42]. Fluorescence was monitored for 3 h and measured with excitation at 485 nm and emission at 538 nm in a Microplate Fluorometer (Biotek Synergy H1).

### 2.6. Statistical Analysis

All the experiments were performed 3 times on the same sample, each one in triplicate. For each subject, normality of data distribution of the replicated measures was confirmed by the Shapiro–Wilk test; after assessing the low intra-experiment and inter-experiment variability and the reproducibility of measures (repeated measures ANOVA, data not shown), each value per subject was calculated as the overall mean of the means of the 3 experiments.

For intra-subjects continuous data, normal distribution was checked using the Shapiro–Wilk test. As the normality assumption was not confirmed, continuous data were reported as median values and interquartile range (IQR) and compared between infertile patients and controls using the Mann–Whitney test for unpaired data. Categorial variables were instead reported as absolute frequencies and percentages and compared between infertile patients and controls using the Fisher exact test.

To investigate the possible association between sperm and systemic redox state and sperm quality, linear regression models were fitted, separately in infertile patients and controls. In addition, multivariable regression models were fitted in infertile patients, to take into account the impact of age and smoking habit on these associations.

All statistical analyses were performed using the software Graph Pad Prism 8 and Stata version 14. A *p*-value of <0.05 was considered statistically significant.

## 3. Results

### 3.1. Subjects

The demographic and clinical characteristics of the 34 idiopathic infertile men and 52 age-matched normozoospermic controls are reported in Table 1.

Semen parameters of the study cohorts are summarized in Table 2. Semen analysis did not show any significant difference in semen volume between infertile patients and controls. Obviously, considering the diagnostic criteria used, significantly lower levels of sperm total count, concentration, and motility were found in infertile patients compared to control subjects (Table 2).

### 3.2. Assessment of Oxidative Stress in Blood and Seminal Fluid

**Figure 1:**
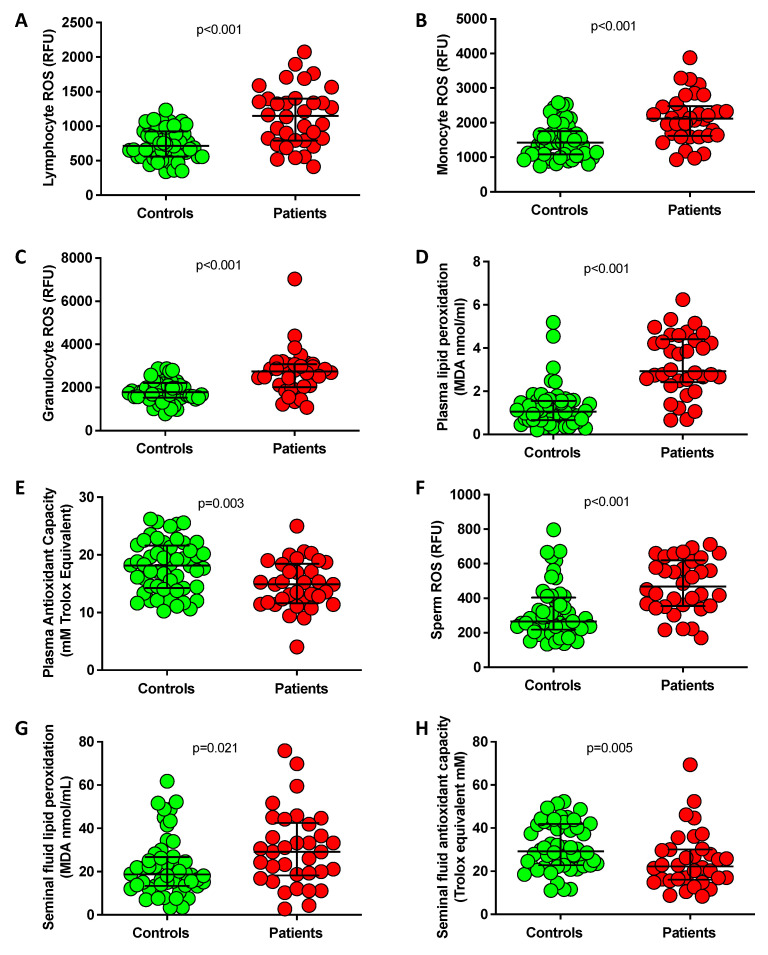
Median (IQR) values and patient-level measures of lymphocyte (**A**), monocyte (**B**) and granulocyte (**C**) ROS production, plasma lipid peroxidation (**D**), plasma total antioxidant capacity (**E**), sperm ROS production (**F**), seminal fluid lipid peroxidation (**G**) and seminal fluid TAC (**H**) in infertile patients (*n* = 34) and controls (*n* = 52). *p*-values are from Mann–Whitney test for unpaired data.

As reported in Figure 1, blood leukocyte subpopulations (lymphocytes, monocytes, and granulocytes) from each infertile patient displayed significantly increased ROS production (Figure 1A–C), and in plasma lipid peroxidation markers were significantly increased (MDA levels, Figure 1D), and total antioxidant capacity (TAC) resulted significantly reduced (Figure 1E) compared to the control group. Moreover, infertile men showed significantly higher sperm ROS levels (Figure 1F) and seminal plasma lipid peroxidation (Figure 1G) together with lower seminal fluid TAC (Figure 1H). All these data are indicative, in infertile patients, of a condition of oxidative stress in the blood, which is mirrored in seminal fluid.

### 3.3. Associations between Investigated Parameters

**Figure 2:**
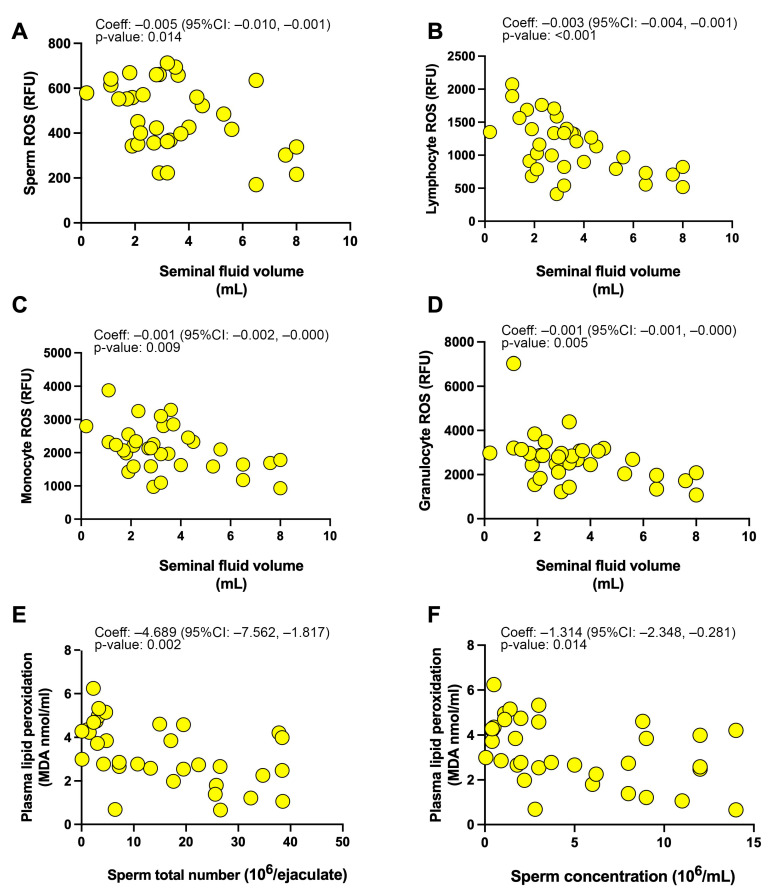
Linear regression coefficient and graphical plots of (**A**) sperm ROS production and seminal fluid volume, (**B**–**D**) leukocyte ROS production and seminal fluid volume, (**E**,**F**), plasma lipid peroxidation and sperm total count (**E**) and sperm concentration (**F**).

We performed linear regression analyses to assess the impact of sperm oxidative stress on sperm function in the 34 infertile patients. As indicated in Table 3a and Figure 2A semen volume was found to significantly decrease for higher values of sperm ROS production, while no relationship emerged between seminal plasma lipid peroxidation and antioxidant capacity and sperm quality parameters (sperm volume, total number, concentration, and motility).

When we explored the possible relationship between systemic oxidative stress parameters and sperm dysfunction (Table 3b and Figure 2B–F), we found that leukocyte ROS production (both by lymphocytes, monocytes, and granulocytes) significantly negatively influenced the seminal volume, and plasma lipid peroxidation significantly negatively influenced both sperm total number and concentration. 

Conversely, when only control subjects were considered (Supplementary Table S1), no significant correlation emerged between sperm or systemic plasma redox parameters and sperm quality features. 

Finally, to take into account the possible confounding role of age and smoking, multivariable regression models were fitted in infertile patients to assess the relationship between sperm and plasma redox state and sperm quality (Supplementary Table S2). Notably, a significant negative relationship was confirmed between sperm and leukocyte ROS production and sperm volume, as well as between plasma lipid peroxidation and sperm total number and concentration.

## 4. Discussion

A growing body of research indicates a link between oxidative stress and male infertility. Although findings about the estimation of redox parameters—more commonly in seminal plasma and less frequently in serum of infertile patients—have been reported [43,44], only a few studies have been focused on the estimation of blood leukocyte ROS production in infertile males. 

In this study, performed in a selected population of idiopathic infertile males, we found that blood redox status is significantly altered both in terms of plasma oxidative stress markers and, prominently, in terms of blood leukocyte ROS production.

One of our main findings is that global blood redox status reflects seminal fluid redox status. In our opinion, this finding can be used as a potentially relevant adjunctive indicator for male infertility diagnosis and management particularly in the case of idiopathic male infertility. Indeed, despite the already reported association between altered sperm quality and semen oxidative damage, men are not usually screened for systemic redox status nor treated to correct a possible redox imbalance. It is well known that sperm function can be highly influenced by alteration in seminal plasma and sperm microenvironment, inducing chemical and structural cellular modifications and modulating sperm fertilization potential [45].

It is well established that ROS are required in the male reproductive system for their sperm fertilization properties, but it has also been clearly shown that ROS overproduction may display deleterious effects on sperm homeostasis leading and/or contributing to male infertility [4,5,6,7,28,46,47,48,49,50,51].

Data from the current literature do not establish if redox status alterations in infertile men reflect active ROS generation by spermatozoa and/or indicate a passive consequence of systemic oxidative stress secondary to lifestyle factors or concomitant habits or diseases (i.e., obesity): this is a key question that needs to be addressed.

The systemic redox status alterations that we revealed in infertile patients can be attributed to different conditions: higher blood leukocyte ROS production, increased plasma lipid peroxidation and reduced plasma total antioxidant levels respect to healthy subjects. Moreover, increased ROS levels in sperm and increased oxidative stress biomarkers in the seminal fluid were also unveiled, indicating a generalized redox imbalance in infertile men.

In line with previous studies suggesting the role of oxidative stress in sperm cell alterations [28,47,52], sperm concentration, total number, and motility were significantly decreased in infertile men compared to healthy subjects.

Agarwal and collaborators underlined the detrimental effects of ROS on sperm motility and morphology: in vitro experiments demonstrated that lipid aldehydes addiction to spermatozoa promoted loss of motility in human sperm cells [50]. Accordingly, our results show that in men with abnormal semen parameters, sperm ROS production is significantly increased compared to controls, and noteworthily, it correlates with sperm total number, concentration, and motility. This finding is in line with previous data and supports the key role of oxidative stress in spermatozoa alterations [51,52,53,54,55,56]. On the contrary, Whittington and co-workers showed no correlation between ROS level and sperm motility, underling that it is still unclear if reduced sperm functional performances are due to lower sperm number or to a direct ROS effect [52].

The assessment of ROS production in human spermatozoa is particularly difficult due to the low levels of sperm-derived ROS compared with those originating from contaminating cell types, particularly neutrophils. This is the reason why flow cytometry techniques were used in our study. This methodology allows the operator to analyze exclusively the sperm population while any contaminating cells, such as precursor germ cells or leukocytes, can be carefully gated out. Moreover, thanks to the fluorometric probes, redox alterations can be significantly correlated to altered sperm function conferring a significant clinical value to this assay [57].

In infertile patients, we found that blood leukocyte ROS production significantly correlated with sperm ROS production, seminal plasma oxidative stress markers, and semen parameters. Additionally, blood oxidative stress markers significantly and positively correlated with semen redox parameters (namely sperm ROS production, seminal plasma lipid peroxidation, and TAC levels), indicating that blood redox alterations reflect sperm dysfunctions in infertile men. In this regard, in agreement with our data, Benedetti and collaborators showed a significant correlation between blood/serum oxidative stress and semen oxidative status in infertile men. In particular, they demonstrated a significant correlation between serum TAC, seminal plasma TAC, and semen parameters [44].

Although a beneficial influence has been generally observed for antioxidants in reversing ROS-induced spermatozoa dysfunction, blood/seminal fluid redox status (in particular ROS production assays) and the use of antioxidants are not routinely used in clinical practice. Indeed, several studies have shown conflicting results about the effects of antioxidants on male fertility [58,59,60]. It is also known that excessive antioxidant intake may induce reductive stress responsible for even detrimental effects on human health [58,59,60].

The reports about the existing association between blood and semen oxidative stress are still limited and controversial, potentially due to different strategies and applied methodologies. Guz and co-workers observed no correlation between blood and semen oxidative status, suggesting the independence of semen redox homeostasis from systemic microenvironment and external factors [61].

Despite the relevant results, some limitations are present in the present study: the study was conducted on a small cohort of patients, and our findings (suggesting blood oxidative stress as a potential predictor of impaired spermatogenesis) need larger studies for validation. Additionally, the causal relationship between oxidative stress and infertility cannot be rigorously explored with this type of study, and temporal variations in redox state or fertility parameters were not explored.

Among the strengths of our study, we want to underline that peripheral blood collection is easier, more practical, less invasive, and less susceptible to sampling inadequacy than semen collection. In addition, recent studies have argued that the lack of standardization in the way routine semen analyses are performed across clinics, often absent of appropriate quality control measures, may limit the accuracy of test interpretations [6]. This can lead to lengthy, stressful, and costly processes for diagnosing male infertility/subfertility, which in turn reduces the likelihood of men undertaking further preconception fertility testing [7].

## 5. Conclusions

In conclusion, our results indicate and confirm the main involvement of oxidative stress in sperm dysfunction, witness that systemic blood oxidative stress reflects semen oxidative status, and suggest blood leukocyte ROS estimation as a new potential and less invasive indicator for male preconception care, especially for idiopathic infertile patients before IVF treatments. Moreover, oxidative stress evaluation may be useful for monitoring new therapeutic approaches based on antioxidant supplementation in order to improve male infertility diagnosis and management.

## Figures and Tables

**Table 1:** Clinical characteristics of 34 idiopathic infertile men and 52 age-matched normozoospermic controls.

Parameter	Controls (*n* = 52)	Idiopathic Infertile Men (*n* = 34)	*p*-Value
Age (years), median (IQR)	39 (36–42)	40 (38–45)	Matching variable
BMI, median (IQR)	24 (22–26)	23 (22–24)	0.059
Smoke, *n* (%)	12 (23.1)	9 (26.5)	0.799

**Table 2:** Semen parameters of 34 idiopathic infertile men and 52 age-matched normozoospermic controls.

Parameter	Controls (*n* = 52) Median (IQR)	Idiopathic Infertile Males (*n* = 34) Median (IQR)	*p*-Value
Semen volume (mL)	3.5 (2.3–4.8)	3.1 (2.1–4.3)	0.344
Sperm concentration (10^6^/mL)	47.0 (30.0–81.0)	3.0 (1.1–8.8)	<0.001
Sperm total number (10^6^/ejaculate)	169.5 (111.0–243.25)	12.0 (3.2–25.8)	<0.001
Progressive motility, PR, (%)	52.5 (40.0–60.0)	30.0 (15.0–40.0)	<0.001
Non-progressive motility, NP (%)	5.0 (5.0–10.0)	10.0 (5.0–20.0)	0.016
Immotile sperm, IM (%)	35.0 (30.0–50.0)	50.0 (45.0–75.0)	<0.001
Normal sperm morphology (%)	6.0 (5.0–6.0)	4.0 (3.0–5.0)	<0.001

**Table 3:** Linear regression analyses between seminal (a) and systemic (b) oxidative stress parameters and sperm volume, total number, concentration, and motility, among the 34 infertile patients.

	Semen Volume	Sperm Total Number	Sperm Concentration	Progressive Sperm Motility
**(a) Seminal redox status**				
Sperm ROS production (RFU)	F(1, 32): 6.73 Coef: −0.005 (−0.010, −0.001) *p*-value: 0.014 *	F(1, 32): 0.00 Coef: +0.000 (−0.030, +0.031) *p*-value: 0.980	F(1, 32): 0.28 Coef: +0.003 (−0.008, +0.013) *p*-value: 0.598	F(1, 32): 0.00 Coef: −0.001 (−0.045, +0.043) *p*-value: 0.973
Seminal plasma lipid peroxidation (MDA nmol/mL)	F(1, 32): 1.59 Coef: −0.025 (−0.066, +0.015) *p*-value: 0.216	F(1, 32): 1.48 Coef: −0.159 (−0.426, +0.107) *p*-value: 0.232	F(1, 32): 0.08 Coef: −0.013 (−0.106, +0.080) *p*-value: 0.777	F(1, 32): 0.18 Coef: +0.080 (−0.309, +0.470) *p*-value: 0.677
Seminal plasma antioxidant capacity (mM Trolox eq.)	F(1, 32): 1.45 Coef: −0.031 (−0.085, +0.022) *p*-value: 0.237	F(1, 32): 2.50 Coef: −0.267 (−0.611, +0.077) *p*-value: 0.124	F(1, 32): 1.10 Coef: −0.062 (−0.181, +0.058) *p*-value: 0.303	F(1, 32): 0.01 Coef: +0.027 (−0.484, +0.539) *p*-value: 0.914
**(b) Systemic redox status**				
Lymphocyte ROS (RFU)	F(1, 32): 15.52 Coef: −0.003 (−0.004, −0.001) *p*-value: <0.001 *	F(1, 32): 1.15 Coef: −0.006 (−0.017, +0.005) *p*-value: 0.292	F(1, 32): 0.28 Coef: +0.001 (−0.003, +0.005) *p*-value: 0.598	F(1, 32): 0.02 Coef: −0.001 (−0.017, +0.015) *p*-value: 0.901
Monocyte ROS (RFU)	F(1, 32): 7.72 Coef: −0.001 (−0.002, −0.000) *p*-value: 0.009 *	F(1, 32): 0.09 Coef: −0.001 (−0.008, +0.006) *p*-value: 0.762	F(1, 32): 0.65 Coef: +0.001 (+0.001, +0.003) *p*-value: 0.425	F(1, 32): 0.90 Coef: −0.005 (−0.014, +0.005) *p*-value: 0.349
Granulocyte ROS (RFU)	F(1, 32): 8.99 Coef: −0.001 (−0.001, −0.000) *p*-value: 0.005 *	F(1, 32): 0.02 Coef: −0.000 (−0.005, +0.004) *p*-value: 0.900	F(1, 32): 2.44 Coef: −0.001 (−0.000, +0.003) *p*-value: 0.128	F(1, 32): 0.67 Coef: −0.003 (−0.009, +0.004) *p*-value: 0.419
Plasma lipid peroxidation (MDA nmol/mL)	F(1, 32): 0.85 Coef:−0.226 (−0.727, +0.275) *p*-value: 0.364	F(1, 32): 11.06 Coef:−4.689(−7.562, −1.817) *p*-value: 0.002 *	F(1, 32): 6.71 Coef: −1.314 (−2.348, −0.281) *p*-value: 0.014 *	F(1, 32): 1.55 Coef:−2.853 (−7.514, +1.808) *p*-value: 0.222
Plasma antioxidant capacity (mM Trolox eq.)	F(1, 32): 0.03 Coef: +0.015 (−0.157, +0.188) *p*-value: 0.857	F(1, 32): 2.72 Coef: −0.883 (−1.973, +0.208) *p*-value: 0.109	F(1, 32): 2.81 Coef: −0.306 (−0.678, + 0.066) *p*-value: 0.103	F(1, 32): 0.36 Coef: −0.478 (−2.097, + 1.140 *p*-value: 0.551

* statistically significant for *p* < 0.05.

## Data Availability

The raw data supporting the conclusions of this article will be made available by the authors, without undue reservation.

## Supplementary Materials

The following supporting information can be downloaded at: https://www.mdpi.com/article/10.3390/antiox12020479/s1, Table S1: Linear regression analyses between seminal (a) and systemic (b) oxidative stress parameters and sperm volume, total number, concentration and motility, among the 52 control subjects, Table S2: Multivariable regression analyses between seminal (a) and systemic (b) oxidative stress parameters and sperm volume, total number, concentration and motility, among the 34 infertile patients, considering in the model also age and smoking habit.

## References

[1] Agarwal A., Baskaran S., Parekh N., Cho C.L., Henkel R., Vij S., Arafa M., Panner Selvam M.K., Shah R. (2021). Male infertility. Lancet.

[2] Wang C., Mbizvo M., Festin M.P., Björndahl L., Toskin I., other Editorial Board Members of the WHO Laboratory Manual for the Examination and Processing of Human Semen (2022). Evolution of the WHO “Semen” processing manual from the first (1980) to the sixth edition (2021). Fertil. Steril..

[3] Zegers-Hochschild F., Adamson G.D., Dyer S., Racowsky C., de Mouzon J., Sokol R., Rienzi L., Sunde A., Schmidt L., Cooke I.D. (2017). The International Glossary on Infertility and Fertility Care. Hum. Reprod..

[4] Pereira S.C., Moreira M.V., Silva B.M., Oliveira P.F., Alves M.G. (2022). Roles of Oxidative Stress in the Male Reproductive System: Potential of Antioxidant Supplementation for Infertility Treatment. Adv. Exp. Med. Biol..

[5] Saleh R.A., Agarwal A. (2002). Oxidative stress and male infertility: From research bench to clinical practice. J. Androl..

[6] Makker K., Agarwal A., Sharma R. (2009). Oxidative stress & male infertility. Indian J. Med. Res..

[7] Agarwal A., Rana M., Qiu E., AlBunni H., Bui A.D., Henkel R. (2018). Role of oxidative stress, infection and inflammation in male infertility. Andrologia.

[8] Cito G., Becatti M., Natali A., Fucci R., Picone R., Cocci A., Falcone P., Criscuoli L., Mannucci A., Argento F.R. (2020). Redox status assessment in infertile patients with non-obstructive azoospermia undergoing testicular sperm extraction: A prospective study. Andrology.

[9] Zini A., Garrels K., Phang D. (2000). Antioxidant activity in the semen of fertile and infertile men. Urology.

[10] Agarwal A., Sharma R.K., Nallella K.P., Thomas A.J., Alvarez J.G., Sikka S.C. (2006). Reactive oxygen species as an independent marker of male factor infertility. Fertil. Steril..

[11] Khosrowbeygi A., Zarghami N. (2007). Levels of oxidative stress biomarkers in seminal plasma and their relationship with seminal parameters. BMC Clin. Pathol..

[12] Aitken J., Fisher H. (1994). Reactive oxygen species generation and human spermatozoa: The balance of benefit and risk. Bioessays.

[13] Janiszewska E., Kokot I., Kmieciak A., Gilowska I., Faundez R., Kratz E.M. (2022). Are There Associations between Seminal Plasma Advanced Oxidation Protein Products and Selected Redox-Associated Biochemical Parameters in Infertile Male Patients? A Preliminary Report. Cells.

[14] Du Plessis S.S., Agarwal A., Halabi J., Tvrda E. (2015). Contemporary evidence on the physiological role of reactive oxygen species in human sperm function. J. Assist. Reprod. Genet..

[15] Kothari S., Thompson A., Agarwal A., du Plessis S.S. (2010). Free radicals: Their beneficial and detrimental effects on sperm function. Indian J. Exp. Biol..

[16] Gosalvez J., Tvrda E., Agarwal A. (2017). Free radical and superoxide reactivity detection in semen quality assessment: Past, present, and future. J. Assist. Reprod. Genet..

[17] Iommiello V.M., Albani E., Di Rosa A., Marras A., Menduni F., Morreale G., Levi S.L., Pisano B., Levi-Setti P.E. (2015). Ejaculate oxidative stress is related with sperm DNA fragmentation and round cells. Int. J. Endocrinol..

[18] Roychoudhury S., Sharma R., Sikka S., Agarwal A. (2016). Diagnostic application of total antioxidant capacity in seminal plasma to assess oxidative stress in male factor infertility. J. Assist. Reprod. Genet..

[19] Sharma R.K., Pasqualotto F.F., Nelson D.R., Thomas A.J., Agarwal A. (1999). The reactive oxygen species-total antioxidant capacity score is a new measure of oxidative stress to predict male infertility. Hum. Reprod..

[20] Agarwal A., Roychoudhury S., Sharma R., Gupta S., Majzoub A., Sabanegh E. (2017). Diagnostic application of oxidation-reduction potential assay for measurement of oxidative stress: Clinical utility in male factor infertility. Reprod. Biomed. Online.

[21] Aitken R.J., Clarkson J.S., Fishel S. (1989). Generation of reactive oxygen species, lipid peroxidation, and human sperm function. Biol. Reprod..

[22] Agarwal A., Saleh R.A., Bedaiwy M.A. (2003). Role of reactive oxygen species in the pathophysiology of human reproduction. Fertil. Steril..

[23] Flesch F.M., Gadella B.M. (2000). Dynamics of the mammalian sperm plasma membrane in the process of fertilization. Biochim. Biophys. Acta.

[24] Shiva M., Gautam A.K., Verma Y., Shivgotra V., Doshi H., Kumar S. (2011). Association between sperm quality, oxidative stress, and seminal antioxidant activity. Clin. Biochem..

[25] Bansal A.K., Bilaspuri G.S. (2010). Impacts of oxidative stress and antioxidants on semen functions. Vet. Med. Int..

[26] Aitken R.J., Baker M.A. (2006). Oxidative stress, sperm survival and fertility control. Mol. Cell Endocrinol..

[27] Venkatesh S., Shamsi M.B., Deka D., Saxena V., Kumar R., Dada R. (2011). Clinical implications of oxidative stress & sperm DNA damage in normozoospermic infertile men. Indian J. Med. Res..

[28] Agarwal A., Virk G., Ong C., du Plessis S.S. (2014). Effect of oxidative stress on male reproduction. World J. Men’s Health.

[29] Bisht S., Faiq M., Tolahunase M., Dada R. (2017). Oxidative stress and male infertility. Nat. Rev. Urol..

[30] Bui A.D., Sharma R., Henkel R., Agarwal A. (2018). Reactive oxygen species impact on sperm DNA and its role in male infertility. Andrologia.

[31] Kamkar N., Ramezanali F., Sabbaghian M. (2018). The relationship between sperm DNA fragmentation, free radicals and antioxidant capacity with idiopathic repeated pregnancy loss. Reprod. Biol..

[32] Agarwal A., Makker K., Sharma R. (2008). Clinical relevance of oxidative stress in male factor infertility: An update. Am. J. Reprod. Immunol..

[33] Vatannejad A., Tavilani H., Sadeghi M.R., Amanpour S., Shapourizadeh S., Doosti M. (2017). Evaluation of ROS-TAC Score and DNA Damage in Fertile Normozoospermic and Infertile Asthenozoospermic Males. Urol. J..

[34] Becatti M., Fucci R., Mannucci A., Barygina V., Mugnaini M., Criscuoli L., Giachini C., Bertocci F., Picone R., Emmi G. (2018). A Biochemical Approach to Detect Oxidative Stress in Infertile Women Undergoing Assisted Reproductive Technology Procedures. Int. J. Mol. Sci..

[35] Sofi F., Dinu M., Pagliai G., Cesari F., Gori A.M., Sereni A., Becatti M., Fiorillo C., Marcucci R., Casini A. (2018). Low-Calorie Vegetarian Versus Mediterranean Diets for Reducing Body Weight and Improving Cardiovascular Risk Profile: CARDIVEG Study (Cardiovascular Prevention With Vegetarian Diet). Circulation.

[36] Becatti M., Emmi G., Silvestri E., Bruschi G., Ciucciarelli L., Squatrito D., Vaglio A., Taddei N., Abbate R., Emmi L. (2016). Neutrophil Activation Promotes Fibrinogen Oxidation and Thrombus Formation in Behçet Disease. Circulation.

[37] Becatti M., Mannucci A., Barygina V., Mascherini G., Emmi G., Silvestri E., Wright D., Taddei N., Galanti G., Fiorillo C. (2017). Redox status alterations during the competitive season in élite soccer players: Focus on peripheral leukocyte-derived ROS. Intern. Emerg. Med..

[38] World Health Organization (2021). WHO Laboratory Manual for the Examination and Processing of Human Semen.

[39] Eggert-Kruse W., Hofsäss A., Haury E., Tilgen W., Gerhard I., Runnebaum B. (1991). Relationship between local anti-sperm antibodies and sperm-mucus interaction in vitro and in vivo. Hum. Reprod..

[40] Fiorillo C., Becatti M., Attanasio M., Lucarini L., Nassi N., Evangelisti L., Porciani M.C., Nassi P., Gensini G.F., Abbate R. (2010). Evidence for oxidative stress in plasma of patients with Marfan syndrome. Int. J. Cardiol..

[41] Whittaker A., Sofi F., Luisi M.L., Rafanelli E., Fiorillo C., Becatti M., Abbate R., Casini A., Gensini G.F., Benedettelli S. (2015). An organic khorasan wheat-based replacement diet improves risk profile of patients with acute coronary syndrome: A randomized crossover trial. Nutrients.

[42] Whittaker A., Dinu M., Cesari F., Gori A.M., Fiorillo C., Becatti M., Casini A., Marcucci R., Benedettelli S., Sofi F. (2017). A khorasan wheat-based replacement diet improves risk profile of patients with type 2 diabetes mellitus (T2DM): A randomized crossover trial. Eur. J. Nutr..

[43] Shamsi M.B., Venkatesh S., Kumar R., Gupta N.P., Malhotra N., Singh N., Mittal S., Arora S., Arya D.S., Talwar P. (2010). Antioxidant levels in blood and seminal plasma and their impact on sperm parameters in infertile men. Indian J. Biochem. Biophys..

[44] Benedetti S., Tagliamonte M.C., Catalani S., Primiterra M., Canestrari F., De Stefani S., Palini S., Bulletti C. (2012). Differences in blood and semen oxidative status in fertile and infertile men, and their relationship with sperm quality. Reprod. Biomed. Online.

[45] Bromfield J.J. (2014). Seminal fluid and reproduction: Much more than previously thought. J. Assist. Reprod. Genet..

[46] Mannucci A., Argento F.R., Fini E., Coccia M.E., Taddei N., Becatti M., Fiorillo C. (2022). The Impact of Oxidative Stress in Male Infertility. Front. Mol. Biosci..

[47] Majzoub A., Agarwal A. (2018). Systematic review of antioxidant types and doses in male infertility: Benefits on semen parameters, advanced sperm function, assisted reproduction and live-birth rate. Arab. J. Urol..

[48] Sabeti P., Pourmasumi S., Rahiminia T., Akyash F., Talebi A.R. (2016). Etiologies of sperm oxidative stress. Int. J. Reprod. Biomed..

[49] Barati E., Nikzad H., Karimian M. (2020). Oxidative stress and male infertility: Current knowledge of pathophysiology and role of antioxidant therapy in disease management. Cell. Mol. Life Sci..

[50] Agarwal A., Sharma R.K., Sharma R., Assidi M., Abuzenadah A.M., Alshahrani S., Durairajanayagam D., Sabanegh E. (2014). Characterizing semen parameters and their association with reactive oxygen species in infertile men. Reprod. Biol. Endocrinol..

[51] Madhu N.R., Sarkar B., Slama P., Jha N.K., Ghorai S.K., Jana S.K., Govindasamy K., Massanyi P., Lukac N., Kumar D. (2022). Effect of Environmental Stressors, Xenobiotics, and Oxidative Stress on Male Reproductive and Sexual Health. Adv. Exp. Med. Biol..

[52] Whittington K., Harrison S.C., Williams K.M., Day J.L., McLaughlin E.A., Hull M.G., Ford W.C. (1999). Reactive oxygen species (ROS) production and the outcome of diagnostic tests of sperm function. Int. J. Androl..

[53] Hosseinzadeh Colagar A., Karimi F., Jorsaraei S.G. (2013). Correlation of sperm parameters with semen lipid peroxidation and total antioxidants levels in astheno- and oligoasheno- teratospermic men. Iran. Red Crescent Med. J..

[54] Bonanno O., Romeo G., Asero P., Pezzino F.M., Castiglione R., Burrello N., Sidoti G., Frajese G.V., Vicari E., D’Agata R. (2016). Sperm of patients with severe asthenozoospermia show biochemical, molecular and genomic alterations. Reproduction.

[55] Aitken R.J. (2017). Reactive oxygen species as mediators of sperm capacitation and pathological damage. Mol. Reprod. Dev..

[56] Dorostghoal M., Kazeminejad S.R., Shahbazian N., Pourmehdi M., Jabbari A. (2017). Oxidative stress status and sperm DNA fragmentation in fertile and infertile men. Andrologia.

[57] Aitken R.J., Smith T.B., Lord T., Kuczera L., Koppers A.J., Naumovski N., Connaughton H., Baker M.A., De Iuliis G.N. (2013). On methods for the detection of reactive oxygen species generation by human spermatozoa: Analysis of the cellular responses to catechol oestrogen, lipid aldehyde, menadione and arachidonic acid. Andrology.

[58] Singh F., Charles A.L., Schlagowski A.I., Bouitbir J., Bonifacio A., Piquard F., Krähenbühl S., Geny B., Zoll J. (2015). Reductive stress impairs myoblasts mitochondrial function and triggers mitochondrial hormesis. Biochim. Biophys. Acta.

[59] Mentor S., Fisher D. (2017). Aggressive Antioxidant Reductive Stress Impairs Brain Endothelial Cell Angiogenesis and Blood Brain Barrier Function. Curr. Neurovasc. Res..

[60] Lamosová D., Juráni M., Greksák M., Nakano M., Vaneková M. (1997). Effect of Rooibos tea (*Aspalathus linearis*) on chick skeletal muscle cell growth in culture. Comp. Biochem. Physiol. C Pharmacol. Toxicol. Endocrinol..

[61] Guz J., Gackowski D., Foksinski M., Rozalski R., Zarakowska E., Siomek A., Szpila A., Kotzbach M., Kotzbach R., Olinski R. (2013). Comparison of oxidative stress/DNA damage in semen and blood of fertile and infertile men. PLoS ONE.

