# Supplementary material for: Blood Leukocyte ROS Production Reflects Seminal Fluid Oxidative Stress and Spermatozoa Dysfunction in Idiopathic Infertile Men
Source: Antioxidants (Basel). 2023 Feb 14;12(2):479. doi: 10.3390/antiox12020479 (PMC9952358; doi:10.3390/antiox12020479)
Supplement: Supplementary file 1 [file antioxidants-12-00479-s001.zip › antioxidants-2161181-supplementary.pdf]

Table S1. Linear regression analyses between seminal (a) and systemic (b) oxidative stress parameters and sperm volume, total number, concentration and motility, among the 52 control subjects.

	Semen Volume	Sperm total number	Sperm concentration	Progressive Sperm Motility (PSR)
**(a) Seminal redox status**				
Sperm ROS production (RFU)	F(1, 50): 1.20 Coef: -0.001 (-0.004,0.001) p-value: 0.279	F(1, 50): 1.33 Coef: -0.105 (-0.286, +0.077) p-value: 0.254	F(1, 50): 0.12 Coef: -0.010 (-0.070, +0.050) p-value: 0.732	F(1, 50): 1.23 Coef: -0.012 (-0.034, +0.0109 p-value: 0.273
Seminal plasma lipid peroxidation (MDA nmol/ml)	F(1, 50): 0.80 Coef: -0.014 (-0.045, +0.017) p-value: 0.375	F(1, 50): 0.71 Coef: -0.876 (-2.957, +1.206) p-value: 0.402	F(1, 50): 0.01 Coef: +0.035 (-0.649, +0.719) p-value: 0.919	F(1, 50): 0.10 Coef: +0.040 (-0.214, +0.295) p-value: 0.753
Seminal plasma TAC (mM Trolox eq.)	F(1, 50): 0.78 Coef: 0.017 (-0.022, 0.056) p-value: 0.382	F(1, 50): 0.37 Coef: +0.793 (-1.833, +3.419) p-value: 0.547	F(1, 50): 0.01 Coef: -0.040 (-0.900, +0.820) p-value: 0.927	F(1, 50): 0.23 Coef: -0.077 (-0.397, +0.243) p-value: 0.630
**(b) Systemic redox status**				
Lymphocyte ROS (RFU)	F(1, 50): 0.34 Coef: -0.001 (-0.001, +0.002) p-value: 0.564	F(1, 50): 0.16 Coef: -0.025 (-0.155, +0.104) p-value: 0.695	F(1, 50): 0.02 Coef: +0.003 (-0.040, +0.045) p-value: 0.893	F(1, 50): 0.22 Coef: -0.004 (-0.019, +0.012) p-value: 0.641
Monocyte ROS (RFU)	F(1, 50): 0.27 Coef: -0.000 (-0.001, +0.001) p-value: 0.604	F(1, 50): 0.00 Coef: -0.001 (-0.057, +0.058) p-value: 0.978	F(1, 50): 0.25 Coef: -0.005 (-0.014, +0.023) p-value: 0.617	F(1, 50): 1.00 Coef: -0.003 (-0.010, +0.003) p-value: 0.322
Granulocyte ROS (RFU)	F(1, 50): 0.72 Coef: -0.000 (-0.000, +0.001) p-value: 0.399	F(1, 50): 0.16 Coef: -0.011 (-0.066, +0.044) p-value: 0.692	F(1, 50): 0.05 Coef: -0.002 (-0.020, +0.016) p-value: 0.822	F(1, 50): 0.19 Coef: -0.001 (-0.008, +0.005) p-value: 0.661
Plasma lipid peroxidation (MDA nmol/ml)	F(1, 50): 0.01 Coef: +0.237 (-0.4426, +0.474) p-value: 0.916	F(1, 50): 0.36 Coef: -9.003 (-39.258, +21.251) p-value: 0.553	F(1, 50): 2.00 Coef: -6.845 (-16.559, +2.869) p-value: 0.163	F(1, 50): 0.32 Coef: -1.032 (-4.771, +2.647) p-value: 0.576
Plasma Antioxidant Capacity (mM Trolox eq.)	F(1, 50): 0.11 Coef: +0.016 (-0.080, +0.112) p-value: 0.739	F(1, 50): 3.30 Coef: +5.688 (-0.601, +11.977) p-value: 0.075	F(1, 50): 1.55 Coef: 1.294 (-0.793, +3.380) p-value: 0.219	F(1, 50): 0.62 Coef: -0.307 (-1.092, +0.477) p-value: 0.435

Table S2. Multivariable regression analyses between seminal (a) and systemic (b) oxidative stress parameters and sperm volume, total number, concentration and motility, among the 34 infertile patients, considering in the model also age and smoking habit.

	**Semen Volume**	**Sperm total number**	**Sperm concentration**	**Progressive Sperm Motility (PSR)**
**(a) Seminal redox status**				
Sperm ROS production (RFU)	F(3, 30): 4.75<br>Coeff: -0.005<br>p-value 0.014*	F(3, 30): 1.63<br>Coeff: 0.006<br>p-value: 0.706	F(3, 30): 0.70<br>Coeff: 0.004<br>p-value: 0.446	F(3,30): 0.22<br>Coeff: 0.004<br>p-value: 0.859
Seminal plasma lipid peroxidation (MDA nmol/ml)	F(3, 30): 2.55<br>Coeff: -0.022<br>p-value: 0.248	F(3, 30): 2.06<br>Coeff: -0.142<br>p-value: 0.269	F(3, 30): 0.51<br>Coeff: -0.009<br>p-value: 0.839	F(3,30): 0.28<br>Coeff: 0.088<br>p-value: 0.656
Seminal plasma TAC (mM Trolox eq.)	F(3, 30): 2.26<br>Coeff: -0.021<br>p-value: 0.424	F(3, 30): 2.19<br>Coeff: -0.212<br>p-value: 0.214	F(3, 30): 0.74<br>Coeff: -0.051<br>p-value: 0.405	F(3, 30): 0.22<br>Coeff: 0.046<br>p-value: 0.862
**(b) Systemic redox status**				
Lymphocyte ROS (RFU)	F(3, 30): 7.48<br>Coeff: -0.003<br>p-value: 0.001*	F(3, 30): 1.64<br>Coeff: -0.002<br>p-value: 0.666	F(3, 30): 0.87<br>Coeff: 0.002<br>p-value: 0.308	F(3, 30): 0.22<br>Coeff: 0.002<br>p-value: 0.837
Monocyte ROS (RFU)	F(3, 30): 5.36<br>Coeff: -0.001<br>p-value: 0.007*	F(3, 30): 1.58<br>Coeff: 0.000<br>p-value: 0.900	F(3, 30): 0.98<br>Coeff: 0.001<br>p-value: 0.250	F(3, 30): 0.37<br>Coeff: -0.004<br>p-value: 0.494
Granulocyte ROS (RFU)	F(3, 30): 6.26<br>Coeff: -0.001<br>p-value: 0.003*	F(3, 30): 1.58<br>Coeff: 0.000<br>p-value: 0.915	F(3, 30): 1.62<br>Coeff: 0.001<br>p-value: 0.083	F(3, 30): 0.35<br>Coeff: -0.002<br>p-value: 0.530
Plasma lipid peroxidation (MDA nmol/ml)	F(3, 30): 2.16<br>Coeff: -0.150<br>p-value: 0.531	F(3, 30): 5.18<br>Coeff: -4.26<br>p-value:0.005*	F(3, 30): 2.43<br>Coeff: -1.24<br>p-value: 0.025*	F(3, 30): 0.61<br>Coeff: -2.60<br>p-value: 0.285
Plasma Antioxidant Capacity (mM Trolox eq.)	F(3, 30): 2.00<br>Coeff: 0.009<br>p-value: 0.912	F(3, 30): 2.86<br>Coeff: -0.929<br>p-value: 0.078	F(3, 30): 1.53<br>Coeff: -0.316<br>p-value: 0.095	F(3, 30): 0.34<br>Coeff: -0.504<br>p-value: 0.539

F(3,30) values are for the entire models, with the three dependent variables (oxidative stress parameters, age and smoking habit). Coefficients and p-values refer to the considered parameters, when adding to the model also age and smoking habit.

*statistically significant for p<0.05
